# Effectiveness and Safety of Rituximab in Filipino Patients With Relapsing-Remitting Multiple Sclerosis: A Real-World Retrospective Cohort Study in a Resource-Limited Setting

**DOI:** 10.7759/cureus.109537

**Published:** 2026-05-24

**Authors:** Alyssa Pauline C Co, Fallen Grace E De la Paz, Myra T Aninang, Ma. Ericka S Del Mundo, Chelsea Mae N Nobleza, Ludwig F Damian

**Affiliations:** 1 Institute for Neurosciences, St. Luke's Medical Center, Quezon City, PHL

**Keywords:** anti-cd20 therapy, demyelinating neurological disorder, philippines, relapsing-remitting multiple sclerosis, rituximab

## Abstract

Background: Multiple sclerosis (MS) is a chronic autoimmune disorder requiring prompt initiation of disease-modifying therapies to mitigate long-term disability. In resource-limited settings like the Philippines, the high cost of approved therapies makes evaluating accessible, off-label high-efficacy treatments like rituximab a clinical priority.

Objective: To evaluate the real-world efficacy and safety of off-label rituximab in patients with relapsing-remitting MS (RRMS) in a tertiary center in the Philippines.

Methods: This single-center retrospective cohort study involved 32 patients with RRMS treated with rituximab between 2017 and 2024. Primary outcomes included the proportion of patients free from clinical relapses, the annualized relapse rate (ARR), and disability progression (as measured by the Expanded Disability Status Scale (EDSS)). Achievement of No Evidence of Disease Activity (NEDA) was assessed, though limited by inconsistent MRI longitudinal data due to financial barriers. The study is further limited by its retrospective design, small sample size, and lack of a comparator arm.

Results: The study included 32 patients (75% female) with a mean age of 25.7 + 8.5 years and a mean baseline EDSS of 1.5 +/- 1.3. Following rituximab initiation, the ARR was 0.55 +/- 0.75. At the two-year follow-up, 75% (n=24) of the cohort achieved NEDA; however, this assessment was based on available clinical and radiological data, as longitudinal MRI was incomplete for a subset of patients due to financial barriers. Disability levels remained stable, with no statistically significant change in mean EDSS from baseline. Rituximab was generally well-tolerated, with mild infusion-related reactions reported in 9.4% (n=3) of patients and no serious adverse events recorded.

Conclusion: In this small observational cohort, rituximab showed potential effectiveness and favorable tolerability, suggesting it may be a viable therapeutic alternative for achieving NEDA in settings where socioeconomic barriers limit access to approved anti-CD20 agents. However, given the lack of a comparator arm and modest sample size, these findings should be interpreted with caution and do not imply therapeutic equivalence to standardized high-efficacy therapies. Larger, prospective comparative studies are warranted.

## Introduction

Multiple sclerosis (MS) is a chronic inflammatory disorder of the central nervous system (CNS) characterized by focal demyelinating lesions in the optic nerves, brainstem, cerebellum, periventricular regions, and spinal cord. It typically begins in adulthood, most commonly between ages 20 and 40, with a female predominance of up to 3:1. There are four major clinical presentations of MS: relapsing-remitting (RRMS), primary-progressive (PPMS), secondary-progressive (SPMS), and progressive-relapsing. Approximately 85% of patients present with an RR course characterized by episodes of neurologic dysfunction followed by complete or partial recovery [1].

Globally, an estimated 1.89 million individuals are living with MS, with more than 62,000 new diagnoses reported in 2021. The global prevalence is 23.9 per 100,000 population and has steadily increased over the past three decades. The highest burden, including prevalence, incidence, disability-adjusted life-years (DALYs), and mortality, has been observed in North America and Western Europe. Countries with the greatest prevalence include Sweden (219 per 100,000), Canada (182), Norway (176), Ireland (163), and the United Kingdom (158) [2].

According to the Global Burden of Disease (GBD) 2021 study, the global prevalence of MS in Asia ranges from approximately 23.9 to 35.9 per 100,000 population and continues to rise. Regional disparities remain pronounced, with countries such as Japan reporting approximately 35,000 cases as of 2024, while Southeast Asia, including the Philippines, remains data-limited [3].

MS is driven by the translocation of autoreactive T-cells across the blood-brain barrier, leading to demyelination, axonal damage, and progressive disability. While the exact etiology remains unclear, the efficacy of newer pharmacotherapies underscores the clinical importance of inhibiting leukocyte entry into the CNS [4].

Modern MS management utilizes a multidisciplinary, coordinated care model. The primary therapeutic goal is to achieve No Evidence of Disease Activity (NEDA), a multifactorial metric defined by the absence of clinical relapses, disability progression, and new or active lesions on MRI [5].

Disability in MS results from cumulative neurological deficits caused by focal inflammation and chronic neurodegeneration. The Kurtzke Expanded Disability Status Scale (EDSS) remains the standard instrument for quantifying this impairment (Kurtzke, 1983). The scale utilizes a quasi-ordinal system ranging from 0.0, representing a normal neurological examination, to 10.0, denoting death attributable to MS complications [6].

The EDSS incorporates findings across eight functional systems, including pyramidal, cerebellar, brainstem, sensory, and visual domains, to generate a composite score of a patient’s status. At lower ranges (EDSS 1.0-3.5), the scale reflects mild-to-moderate impairment while patients remain fully ambulatory. However, the scale is characterized by a heavy reliance on ambulatory milestones at the mid-range; for instance, EDSS 6.0 is strictly defined by the requirement of a unilateral walking aid to traverse 100 meters [7]. A notable limitation of the EDSS is its focus on physical mobility, which often overlooks non-ambulatory factors such as cognition, mental health, and fatigue.

Effective management focuses on early preservation of neurological reserve rather than reactive symptom control. The introduction of B-cell-depleting therapies, such as ocrelizumab and rituximab, has shifted the treatment landscape. By targeting the CD20 antigen on B lymphocytes, these agents suppress the inflammatory processes driving both clinical relapses and subclinical disease progression [8].

In the Philippines, the clinical goal of preventing irreversible disability is frequently complicated by significant economic barriers to high-efficacy care. While ocrelizumab is recognized globally for its superior ability to slow progression in both relapsing and primary progressive forms of MS, its utilization in the Philippine healthcare setting remains severely limited due to its prohibitive cost and a lack of comprehensive insurance coverage.

Consequently, many Filipino neurologists turn to rituximab as a strategic, cost-effective alternative. Although used "off-label" for MS, rituximab shares a similar mechanism of action by targeting CD20-positive B-cells to suppress CNS inflammation. This off-label use is often a necessity in local practice, providing a vital pathway for patients to access B-cell-depleting therapy that would otherwise be financially out of reach. By leveraging rituximab, clinicians can still aim to close the "window of opportunity" and mitigate long-term axonal loss, tailoring the global standard of high-efficacy treatment to the specific socio-economic realities of the Philippine patient population.

Expanding upon recent clinical evidence, Granqvist et al. (2018) demonstrated that rituximab significantly outperformed conventional disease-modifying therapies (DMTs) in newly diagnosed RRMS, reporting markedly lower clinical relapse rates and reduced neuroradiologic activity [9]. This high-efficacy profile is particularly relevant in the Philippines, where cost-prohibitive access to ocrelizumab necessitates the use of more affordable B-cell-depleting agents. Consequently, our study aimed at evaluating the efficacy and safety of rituximab in patients with RRMS in the local setting, seeking to validate its clinical utility within the unique socio-economic and demographic context of the Filipino population.

Despite the established efficacy of anti-CD20 therapies globally, there is a lack of real-world evidence regarding their long-term use in Southeast Asian populations facing significant socioeconomic barriers. This study addresses this gap by evaluating the safety and effectiveness of rituximab in Filipino patients with RRMS. By analyzing attainment of NEDA and long-term disability outcomes, this research seeks to establish an evidence-based approach that optimizes treatment efficacy within local resource constraints.

## Materials and methods

Study design and participant selection

This single-center retrospective cohort study was conducted at St. Luke’s Medical Center (Global City and Quezon City, Philippines) following approval by the Institutional Review Board (IRB). We reviewed medical records of patients diagnosed with RRMS according to the 2017 revised McDonald criteria who received off-label rituximab therapy between January 2017 and January 2024 [10]. Eligibility required a minimum of three months of clinical follow-up post-rituximab initiation. Patients receiving rituximab for non-MS indications or those with incomplete medical records were excluded. As a retrospective pilot study, no formal a priori power calculation was performed; the sample size was determined by the available clinical registry. We acknowledge a potential risk of selection bias inherent in the retrospective recruitment of patients from a tertiary referral center.

Treatment protocol and monitoring

The standardized rituximab regimen consisted of a 1000-mg intravenous loading dose (administered as two 500-mg infusions separated by 14 days), followed by 500-mg maintenance infusions every six months. Clinical follow-up occurred at three-to-six-month intervals. B-cell depletion (CD19/CD20 counts) was monitored to guide clinical decisions where feasible, though acquisition was non-uniform due to socioeconomic constraints.

Data acquisition and clinical assessment

Comprehensive data were extracted from institutional electronic health records, encompassing demographic profiles (age and sex) and clinical characteristics, including age at diagnosis, total disease duration, and relevant comorbidities. We also documented treatment-specific factors such as age at rituximab initiation, total duration of therapy, previous exposure to DMTs, and clinical indications for switching to rituximab.

Disability was quantified using the Kurtzke EDSS. As part of routine institutional practice, patients underwent clinical evaluations and 1.5-Tesla MRI, including pre- and post-gadolinium sequences, every six to 12 months.

The standardized MS protocol included axial T2-weighted, fluid-attenuated inversion recovery (FLAIR), and pre- and post-gadolinium T1-weighted sequences with a 3-mm slice thickness. Contrast (gadobenate dimeglumine or equivalent) was administered at a standard dose of 0.1 mmol/kg. Active radiologic disease was defined by the presence of new or enlarging T2-hyperintense lesions or T1-gadolinium-enhancing lesions. Images were reviewed by board-certified neuroradiologists, and to ensure diagnostic robustness, readers were blinded to specific clinical outcome timelines. Interobserver discrepancies were resolved through consensus adjudication.

Outcomes

The efficacy of rituximab was evaluated through four primary endpoints designed to capture both clinical and radiological disease stability. These included: the proportion of patients who remained entirely free from clinical relapses throughout the study period; the annualized relapse rate (ARR); the proportion of patients without disability progression, as quantified by stable or improved scores on the EDSS; and the proportion of patients achieving NEDA.

For the purposes of this study, NEDA was defined as a composite status reflecting the total absence of clinical relapses, confirmed disability progression, and radiologic disease activity, characterized by new T2-weighted or gadolinium-enhancing lesions on MRI. In instances where follow-up MRI data were unavailable due to institutional or financial constraints, NEDA classification was adjudicated based on a comprehensive review of available longitudinal clinical data. Safety outcomes were assessed by documenting all reported adverse events, with a particular emphasis on the incidence and severity of acute infusion-related reactions.

Statistical analysis

Statistical analyses were conducted using IBM SPSS Statistics for Windows, Version 26 (Released 2018; IBM Corp., Armonk, New York, United States), with a two-sided p-value of < 0.05 established as the threshold for statistical significance. The normality of continuous variables was evaluated using the Shapiro-Wilk test. Data were summarized as mean ± standard deviation (SD) or median with range for continuous variables, while categorical data were expressed as absolute frequencies and percentages.

ARR was calculated as the total number of relapses divided by the total person-years of follow-up. Comparative analysis of pre- and post-treatment ARR and EDSS scores was performed using the Wilcoxon signed-rank test. To improve analytical robustness, the probability of remaining relapse-free was estimated using Kaplan-Meier survival analysis. Binary logistic regression was utilized to evaluate factors associated with achieving NEDA, providing odds ratios (OR) and 95% confidence intervals (CI). A p-value of < 0.05 was considered statistically significant. No formal correction for multiple comparisons was applied, given the exploratory nature of the study.

Ethical considerations

This study received formal approval from the St. Luke’s Medical Center Institutional Ethics Review Committee (Protocol No. RPC-095-03-24). The research was conducted in strict adherence to the ethical principles outlined in the Declaration of Helsinki (2013) and complied with the International Council for Harmonization Good Clinical Practice (ICH-GCP) guidelines. In compliance with the Philippine Data Privacy Act, patient confidentiality was rigorously maintained; all collected data were fully anonymized and remained accessible only to authorized study personnel.

## Results

Baseline demographics and clinical characteristics

A total of 32 patients with RRMS who received rituximab at St. Luke’s Medical Center (Global City and Quezon City) between January 2017 and January 2024 were included in the study. The cohort was predominantly female (n=24, 75%), with a mean age of 25.7 ± 8.5 years. The mean duration from initial symptom onset to definitive diagnosis was 4.5 ± 14.5 months. At baseline, the mean EDSS score was 1.5 ± 1.3 (range 0-5), indicating a relatively mild degree of neurological impairment at the time of treatment initiation. The comprehensive demographic and clinical profiles are summarized in Table 1.

**Table 1:** Demographic and clinical characteristics of RRMS patients (n=32) Baseline demographic and clinical characteristics of patients with relapsing-remitting multiple sclerosis (RRMS) treated with rituximab (RTX). Data are expressed as mean +/- standard deviation (SD), absolute frequency (n), and percentage (%), or range where indicated. The p-value for Expanded Disability Status Scale (EDSS) represents a paired comparison between baseline and post-treatment scores using the Wilcoxon signed-rank test. Statistical significance is defined as p < 0.05. Significant p-values are denoted with an asterisk (*).

Variable	Value	P-value
Age, mean +/- SD (years)	25.7 +/- 8.5	—
Female, n (%)	24 (75%)	—
Disease duration (months), mean +/- SD	4.5 +/- 14.5	—
Clinical Presentation		
Visual disturbance, n (%)	12 (38%)	—
Numbness, n (%)	8 (26%)	—
Weakness, n (%)	8 (24%)	—
Back pain, n (%)	2 (6%)	—
Gait imbalance, n (%)	2 (6%)	—
MRI Lesion Location		
Periventricular, n (%)	12 (37%)	—
Juxtacortical, n (%)	8 (25%)	—
Optic nerve, n (%)	5 (16%)	—
Cortical, n (%)	4 (12%)	—
Spinal cord, n (%)	3 (10%)	—
Relapse and Treatment History		
Relapses (past 2 years), mean +/- SD	1.1 +/- 1.0	—
Time to RTX initiation (months), mean +/- SD	3.7 +/- 2.8	—
Disability Outcomes		
Baseline EDSS, mean +/- SD (range)	1.5 +/- 1.3 (0–5)	0.096
Post-RTX EDSS, mean +/- (range)	0.8 + 1.5 (0–5)	

Symptomatology and neuroimaging profiles

**Figure 1:**
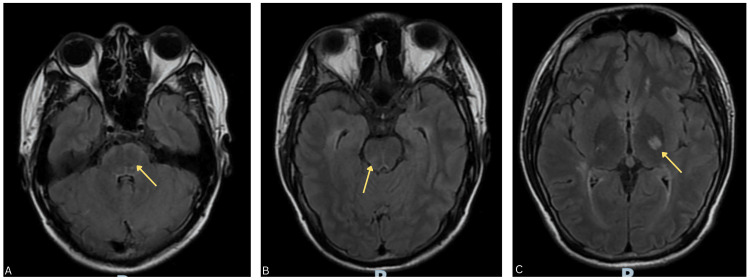
Characteristic brain MRI findings in a 32-year-old male with multiple sclerosis Axial fluid-attenuated inversion recovery (FLAIR) sequences demonstrating typical focal hyperintense demyelinating plaques (indicated by yellow arrows). A: hyperintense lesion located in the left pons; B: lesion in the right cerebral peduncle (midbrain); and C: lesion involving the left thalamus and adjacent deep white matter.

**Figure 2:**
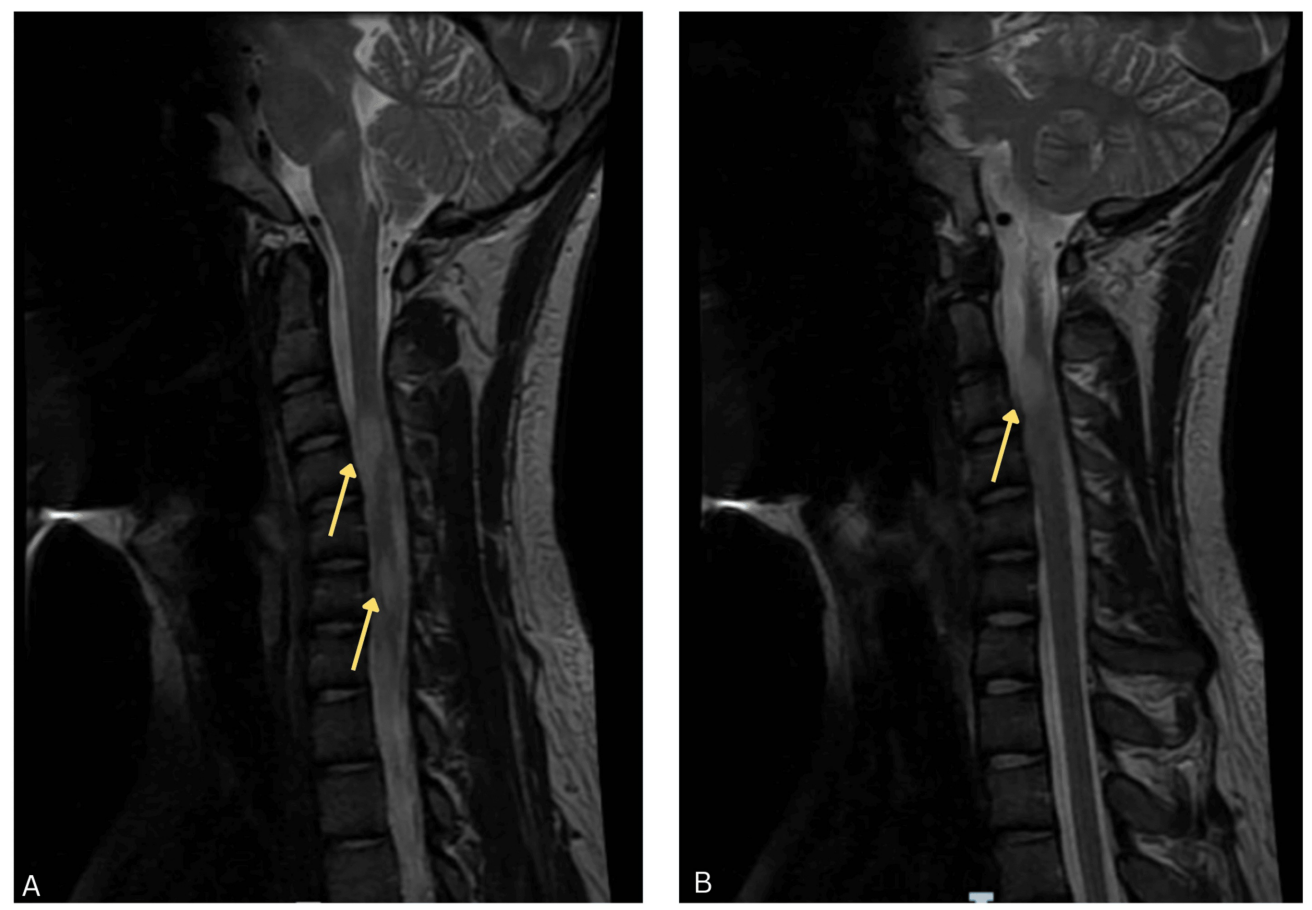
Cervical spine MRI of a 32-year-old male with multiple sclerosis (MS) A, B: sagittal T2-weighted sequences showing multifocal, hyperintense intramedullary lesions within the cervical spinal cord (yellow arrows). These characteristic short-segment plaques are classic manifestations of spinal cord demyelination in MS.

Visual disturbance was the most frequent presenting symptom (n=12, 38%), followed by sensory numbness (n=8, 26%), motor weakness (n=8, 24%), back pain (n=2, 6%), and gait imbalance (n=2, 6%). Baseline magnetic resonance imaging (MRI) revealed a distribution of demyelinating lesions characteristic of MS, with periventricular involvement being the most prevalent (n=12, 37%). Other involved regions included juxtacortical (n=8, 25%), optic nerve (n=5, 16%), cortical (n=4, 12%), and spinal cord (n=3, 10%) locations. MRI details of patients with MS are captured in Figures 1, 2.

Treatment response and disability outcomes

Prior to the initiation of rituximab, the mean number of relapses within the preceding two-year period was 1.1 ± 1.0 (range 0-4). The mean interval from diagnosis to rituximab initiation was 3.7 ± 2.8 months. Following treatment, the mean EDSS score demonstrated a downward trend from a baseline of 1.5 ± 1.3 to 0.8 ± 1.5 (range 0-7) at final follow-up (p=0.096). This suggests a stabilization or numerical improvement in physical disability across the cohort during the observation period.

B-cell depletion and clinical efficacy

Rituximab administration resulted in a profound and sustained depletion of peripheral B-cell markers. The mean CD19+ count decreased significantly from a baseline of 317.3 ± 205.5 cells/µL to 53.8 ± 39.5 cells/µL after the first infusion, reaching 28.0 ± 29.4 cells/µL by the fourth infusion (p < 0.001*). Similarly, CD20+ counts dropped from 247.5 ± 216.0 cells/µL at baseline to 31.8 ± 31.6 cells/µL following the fourth infusion (p < 0.001*), confirming effective and continuous B-cell suppression throughout the treatment course. These longitudinal trends are summarized in Table 2.

**Table 2:** Longitudinal trends in CD19 and CD20 lymphocyte counts (n=32) CD19 and CD20 counts are expressed as cells/µL. p-values represent paired comparisons between baseline and respective post-treatment time points using the Wilcoxon signed-rank test. Statistical significance is defined as p < 0.05; highly significant values (p < 0.001) are denoted with an asterisk (*). RTX: rituximab

Time Point	CD19 Count (cells/µL)	p-value	CD20 Count (cells/µL)	p-value
Baseline (mean +/- SD)	317.3 +/- 205.5	Reference	247.5 +/- 216.0	Reference
Post-1st RTX (mean +/- SD)	53.8 +/- 39.5	< 0.001*	61.2 +/- 42.9	< 0.001*
Post-4th RTX (mean +/- SD)	28.0 +/- 29.4	< 0.001*	31.8 +/- 31.6	< 0.001*

The ARR during follow-up was 0.55 ± 0.75. At two years, 75% (n=24) of patients achieved NEDA. Rituximab infusions were generally well tolerated. Mild infusion-related rash occurred in three of 32 patients (9.4%) and resolved with a reduction in infusion rate. No serious adverse events were observed.

Factors associated with treatment response

Bivariate analyses were conducted to identify baseline predictors of clinical success, specifically the attainment of NEDA. Baseline demographic and clinical variables, including age (p=0.76), sex (p=1.00), duration from symptom onset to diagnosis (p=0.47), baseline EDSS (p=0.94), and previous exposure to immunosuppressive therapies (p=0.37), demonstrated no statistically significant association with NEDA achievement, with 95% CIs encompassing the null value for all variables.

Notably, only the frequency of pre-treatment relapses was found to be a significant predictor of the likelihood of achieving NEDA (p < 0.005*). While the lack of complete longitudinal paired data precluded a formal comparative statistical analysis of lymphocyte subsets, descriptive trends revealed a marked and consistent reduction in both CD19 and CD20 levels across all post-treatment evaluation points (Table 3).

**Table 3:** Bivariate analysis of factors associated with NEDA achievement (n=32) p-values were derived from bivariate analyses (Pearson’s correlation or non-parametric equivalent) exploring associations with the achievement of No Evidence of Disease Activity (NEDA). Statistical significance is defined as p < 0.05. Highly significant predictors are denoted with an asterisk (*).

Baseline Variable	p-value
Age	0.76
Sex	1.00
Duration from symptom onset to diagnosis	0.47
Baseline Expanded Disability Status Scale (EDSS)	0.94
Prior immunosuppressive therapies	0.37
Number of pre-treatment relapses	< 0.005*

## Discussion

This study provides a real-world evaluation of off-label rituximab use for RRMS within a tertiary healthcare setting in the Philippines. Our findings suggest that rituximab may contribute to reduced relapse rates and clinical disability stability, with a calculated 75% of patients achieving NEDA over a two-year period. These results align with the expanding global consensus regarding the high efficacy of B-cell-depleting therapies in managing MS pathophysiology, though they must be interpreted within the context of our study's observational nature [11,12].

In our cohort, rituximab was associated with a reduction in the ARR and a trend toward disability stability. While the downward trend in mean EDSS scores did not reach statistical significance, this is a common observation in observational MS studies where inflammatory markers typically respond more rapidly to anti-CD20 therapy than clinical disability [9,11-13]. However, the interpretation of disability stability in this cohort is limited by the relatively low baseline EDSS (mean 1.5), where "pseudo-stability" may occur, as detecting progression in mildly disabled patients often requires longer follow-up or more sensitive metrics. Furthermore, the 75% NEDA achievement likely represents an optimistic estimate, as NEDA classification was adjudicated based on available clinical data in instances where longitudinal MRI surveillance was incomplete due to financial barriers.

The biological activity of the treatment was supported by the sustained reduction in CD19+ and CD20+ lymphocyte counts [14]. Interestingly, our finding that the number of pre-treatment relapses was the only significant predictor of NEDA achievement suggests that patients with higher baseline inflammatory activity may derive the most measurable benefit from rapid B-cell depletion. Nevertheless, these findings remain preliminary given our modest sample size and the absence of a comparator arm, which precludes definitive claims of therapeutic superiority or equivalence to approved agents.

Methodological considerations, including the retrospective design, introduce the risk of survivorship bias, as the cohort likely consists of patients who tolerated and responded well enough to the medication to remain in follow-up. Safety remains a paramount concern for long-term immunosuppression. In our study, rituximab was well-tolerated, with only mild infusion-related reactions. While chronic B-cell depletion carries theoretical risks of hypogammaglobulinemia, the safety profile observed here supports the short-to-medium-term viability of this regimen in a local setting [10,15,16].

A critical takeaway from our study is the role of rituximab as a high-efficacy, cost-effective alternative in low-to-middle-income countries (LMICs). While newer anti-CD20 agents like ocrelizumab have gained regulatory approval, their prohibitive costs often render them inaccessible in the Philippines. Our data suggest that off-label rituximab provides a practical pathway to achieving favorable clinical outcomes within resource-constrained environments. Given that data on MS in Asian populations remain relatively limited, our findings provide valuable local evidence supporting the utility of rituximab in a Filipino population while highlighting the need for larger, prospective comparative trials to validate these observational trends.

Limitations of the study

This study has several limitations that necessitate a cautious interpretation of the findings, framing this work as an exploratory real-world observational study rather than a definitive efficacy analysis.

First, as a single-center retrospective analysis with a modest sample size (n=32), the results may lack broader generalizability. The retrospective design and recruitment from a tertiary center introduce potential selection and survivorship bias, as the cohort inherently represents patients who tolerated the treatment well enough to remain in long-term follow-up.

Second, the absence of a comparator arm precludes direct efficacy comparisons with approved high-efficacy DMTs. Third, due to the socioeconomic realities of our setting, longitudinal follow-up was subject to missing data. Specifically, non-uniform B-cell monitoring and inconsistent MRI surveillance occurred due to patient financial constraints. This imaging missingness potentially leads to an overestimation of NEDA achievement, as subclinical radiologic activity may have gone undetected in a subset of patients.

Furthermore, the relatively mild baseline disability of the cohort (mean EDSS 1.5) introduces the risk of "pseudo-stability," where standard clinical metrics may be insufficiently sensitive to capture subtle neurological progression over the observation period. Finally, while binary logistic regression was applied, the limited statistical power inherent to our small cohort restricted the feasibility of robust multivariate analysis, limiting our ability to fully adjust for all potential confounding factors.

## Conclusions

Our study demonstrates that off-label rituximab is a highly effective and well-tolerated strategy for managing RRMS in a Filipino cohort. With 75% of patients achieving clinical and radiological stability (NEDA) over a two-year period, these results substantiate rituximab as a vital, high-efficacy alternative in resource-limited settings where socioeconomic barriers frequently restrict access to newer, approved anti-CD20 agents.

While we acknowledge the observational nature of the data and the inherent limitations of a retrospective design, our findings provide compelling real-world evidence for rituximab’s role in stabilizing disease activity and bridging the critical treatment gap in the Philippines. Larger, prospective comparative studies remain essential to definitively validate these trends and to evaluate the long-term impact of this regimen on disability accrual in Asian populations.
